# Effect of Individual Virtual Reality Cognitive Training Programs on Cognitive Function and Depression in Middle-Aged Women: Randomized Controlled Trial

**DOI:** 10.2196/48912

**Published:** 2023-10-25

**Authors:** Du-Ri Kim, EunSoo Moon, Myung-Jun Shin, Yeong-Ae Yang, Jong-Hwan Park

**Affiliations:** 1 Health Convergence Medicine Laboratory, Biomedical Research Institute, Pusan National University Hospital Busan Republic of Korea; 2 Department of Rehabilitation Science, Graduate School, Inje University Gimhae Republic of Korea; 3 Department of Psychiatry, Pusan National University School of Medicine Yangsan Republic of Korea; 4 Department of Psychiatry, Pusan National University Hospital Busan Republic of Korea; 5 Department of Rehabilitation Medicine, Pusan National University School of Medicine Yangsan Republic of Korea; 6 Department of Rehabilitation Medicine, Pusan National University Hospital Busan Republic of Korea; 7 Department of Occupational Therapy, College of Biomedical Science and Engineering, Inje University Gimhae Republic of Korea; 8 Institute of Aged Life Redesign, Inje University Gimhae Republic of Korea

**Keywords:** cognitive function, depression, middle aged, virtual reality, women

## Abstract

**Background:**

Given the increasing incidence of early-onset Alzheimer disease, strategies for early diagnosis and swift treatment interventions are crucial for mitigating cognitive problems in women and middle-aged individuals who face a high risk of cognitive impairment.

**Objective:**

This study aimed to assess the effectiveness of individual cognitive training programs based on virtual reality (VR), a nonpharmacological intervention, on cognitive function and depression in middle-aged women at risk of cognitive impairment. It used VR technology, which has recently been recognized as a promising tool.

**Methods:**

We administered a VR-based cognitive training program for 30 minutes daily, twice a week, for 12 weeks (24 sessions). This study included middle-aged women residing in older adults’ welfare facilities in G-gu, Busan, from May to August 2021. A total of 60 participants were randomly divided into the training (n=30) and control (n=30) groups. Cognitive and depressive functions were assessed using the Korean versions of the Montreal Cognitive Assessment (K-MoCA), Digit Span Test (DST), Korean-Color Word Stroop Test (K-CWST), and Short Form of the Geriatric Depression Scale (SGDS-K) before the intervention. The training group underwent a VR-based cognitive training program, whereas the control group was educated to maintain regular daily activities. The same assessments were performed 12 weeks after treatment.

**Results:**

A comparison of the mean scores before and after K-MoCA in the training group revealed a significant increase from 24.87 (SD 2.62) to 27.50 (SD 1.70; *P*<.01), indicating substantial cognitive improvement. Similarly, the mean DST forward scores increased significantly from 6.97 (SD 1.10) to 7.90 (SD 1.18; *P*<.01), suggesting enhanced short-term auditory memory and attention. The mean DST backward scores also showed a significant improvement from 4.10 (SD 0.71) to 4.77 (SD 1.2; *P*=.01). Notably, the mean SGDS-K scores decreased significantly from 3.97 (SD 2.51) to 2.13 (SD 1.87; *P*<.01), indicating a reduction in depression within the training group.

**Conclusions:**

The VR-based cognitive training programs significantly enhanced cognitive function and reduced depression in middle-aged women. Consequently, these programs are considered beneficial nonpharmacological cognitive training interventions for middle-aged women at high risk of cognitive impairment.

**Trial Registration:**

UMIN Clinical Trials Registry UMIN000049752; https://tinyurl.com/z5du989z

## Introduction

Cognitive aging begins in middle-aged women, leading to a decline in processing speed and memory, particularly in postmenopausal women. Additionally, the risk of estrogen loss and neurological decline following menopause is increased during this period, subsequently elevating the susceptibility of middle-aged women to Alzheimer disease compared with men [1,2]. Alzheimer disease is the most prevalent form of dementia and a significant global public health concern [3].

Recent investigations into early-onset Alzheimer disease (EOAD) have revealed a lack of domestic research on nonpharmaceutical approaches to EOAD, underscoring the pressing need for further research owing to the insufficient verification of long-term effects [4]. Given the increasing annual incidence of EOAD, there is an imperative demand for strategies geared toward early diagnosis and swift treatment interventions to combat cognitive issues in middle-aged individuals and women, both of whom face heightened risks of cognitive impairment. Recent discussions have emphasized the need for research aimed at evaluating the effectiveness of nonpharmacological cognitive interventions [5]. Cognitive training and behavioral interventions that do not involve pharmaceutical components have been shown to be effective in alleviating behavioral symptoms such as depression, apathy, wandering, sleep disturbances, and aggression. These interventions play a crucial role in preserving cognitive function and reducing cognitive decline [6]. The Lancet Commission’s 2020 report examined the connection between depression and cognitive impairment [7]. This highlights the link between depression and the development of dementia through various psychological and physiological mechanisms, particularly during the early stages of dementia. Therefore, engaging in cognitive activities during middle age is recommended to reduce the risk of dementia through behavioral changes.

Novel virtual reality (VR)–based technologies have emerged to enhance the efficacy of cognitive training. This technology immerses the user in virtual environments and transmits various visual, auditory, and sensory stimuli through a head-mounted display (HMD) [8]. VR is a compelling approach for individuals with cognitive impairment, where regular training yields increased effectiveness, positioning it as a promising avenue for dementia treatment [9].

VR-based cognitive training harnesses multiple senses, uses engaging gaming formats, drives strong motivation, and improves cognitive function. These interventions have demonstrated favorable outcomes, particularly in memory, executive function, and various cognitive domains [10,11]. Nonetheless, given the individualized nature of age-related cognitive function decline, it is imperative to develop individually tailored cognitive training programs.

Consequently, this study sought to ascertain the effectiveness of VR-based individual cognitive training programs, which fall under the umbrella of nonpharmacological interventions, in addressing cognitive function and depression among middle-aged women at high risk of cognitive impairment. This endeavor has leveraged VR technology, which has recently been recognized as a promising tool in this context.

## Methods

### Recruitment

For 12 weeks, from May to August 2021, the objectives and methodology of this study were presented at 3 older adults’ welfare facilities situated in G-gu. The recruitment of participants involved the issuance of recruitment notices to middle-aged women 40-65 years old who expressed a voluntary willingness to participate and met specific selection criteria. The inclusion criteria included individuals who (1) visited organizations and centers, (2) had no physical disabilities, (3) were able to read and write for evaluation, (4) had the ability to understand the purpose of this test and voluntarily agreed by signing the consent form, or (5) had a Mini-Mental State Examination (MMSE) score of 24-28 points. Patients (1) who had difficulty wearing an HMD and operating controller equipment owing to severe or unstable physical conditions and who had medical conditions that may interfere with the completion of clinical trials, (2) with other degenerative brain diseases or mental illnesses such as depression, (3) with drug and alcohol addiction, and (4) with hearing and vision impairment that could not be evaluated effectively were excluded. After determining the appropriate number of participants, based on the effect size calculated in previous studies involving VR-based cognitive training, only 34 patients in each group exhibited significant improvements in cognitive function [12].

### Study Design

**Figure 1:**
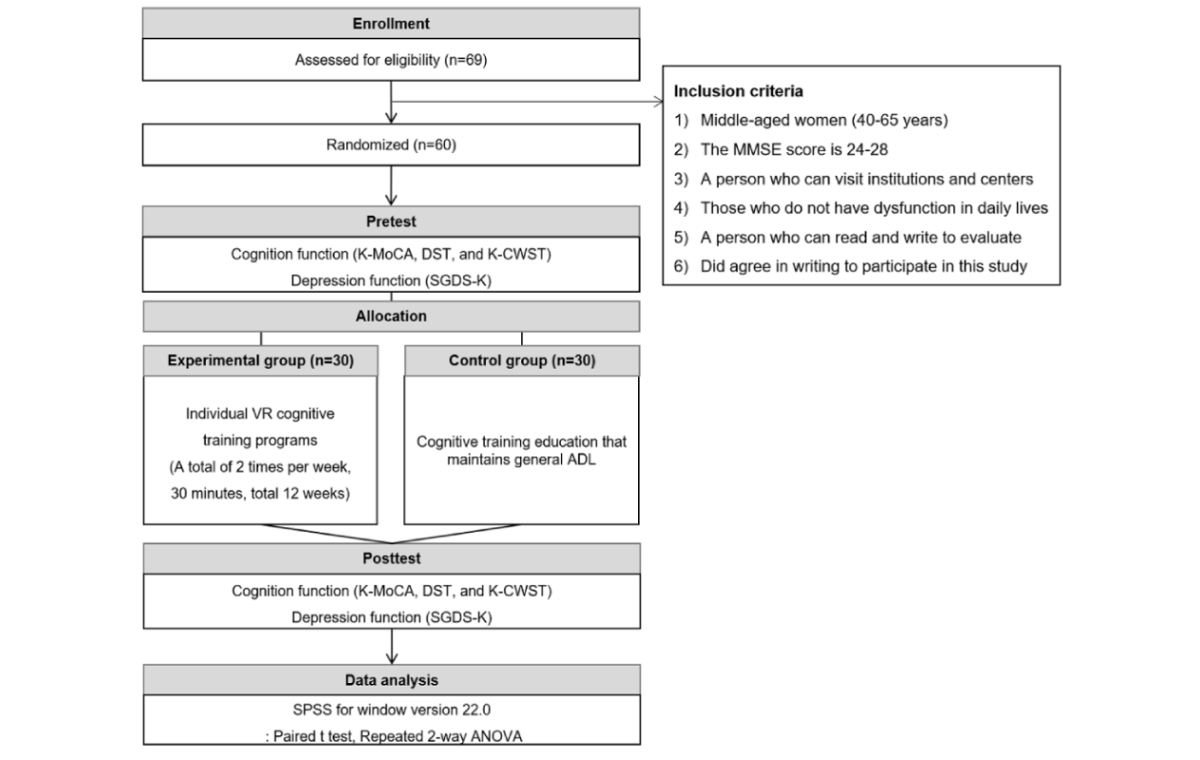
Flowchart of the process of selecting the participants and measurement methods. ADL: activities of daily living; DST: Digit Span Test; K-CWST: Korean-Color Word Stroop Test; K-MoCA: The Korean version of the Montreal Cognitive Assessment; MMSE: Mini-Mental State Examination; SGDS-K: The Korean version of the Short Form of Geriatric Depression Scale; VR: virtual reality.

This study used a pre-post experimental design with a randomized control group to assess the impact of VR-based individual cognitive training programs on cognitive function and depression in middle-aged women. Middle-aged women between the ages of 40 and 65 years who voluntarily expressed interest in participating and met the specified selection criteria were recruited. Participants were randomly allocated to either the experimental group (n=30) or the control group (n=30) using block randomization facilitated by SAS programming (SAS Institute), maintaining an equal 1:1 ratio. The VR cognitive training program was structured as a comprehensive cognitive function training regimen. The experimental group participated in a VR-based individual cognitive training program twice a week for 12 weeks, with each session lasting 30 minutes. The participants in the control group were instructed to continue their regular daily activities and cognitive training throughout the study (Table 1). Assessments were performed before the initiation of the VR-based training program (baseline) and after the intervention (follow-up). The procedural flow of the study is shown in Figure 1. This trial was registered under the identifier UMIN000049752 in the UMIN Clinical Trial Registration database. All educational and training activities in this study were conducted by researchers with a strong understanding of the equipment and occupational therapy and extensive experience in operating the equipment. To assess the effects of VR-based cognitive training programs on cognitive function and depression in middle-aged women, several standardized tests were used, including the Korean version of the Montreal Cognitive Assessment (K-MoCA), Digit Span Test (DST), Korean Color Word Stroop Test (K-CWST), and the Korean version of the Short Form of Geriatric Depression Scale (SGDS-K; Figure 1).

**Table 1:** Cognitive training education given to the control group.

Cognitive domain	Duration (weeks)	Cognitive training education during activities of daily living
Memory	1-3	Habit of taking notes on important matters Save it on your phone Habit of putting things in the same place
Attention	4-6	Focus on one thing When concentrating, move to a quiet place and deal with it
Language	7-9	Prepare in advance and practice loudly before the meeting Listen carefully and follow the story
Mix and executive function	10-12	Mental arithmetic Memorizing well-known sayings Eat food that helps your brain

### Measurements

#### VR-Based Cognitive Evaluation

**Figure 2:**
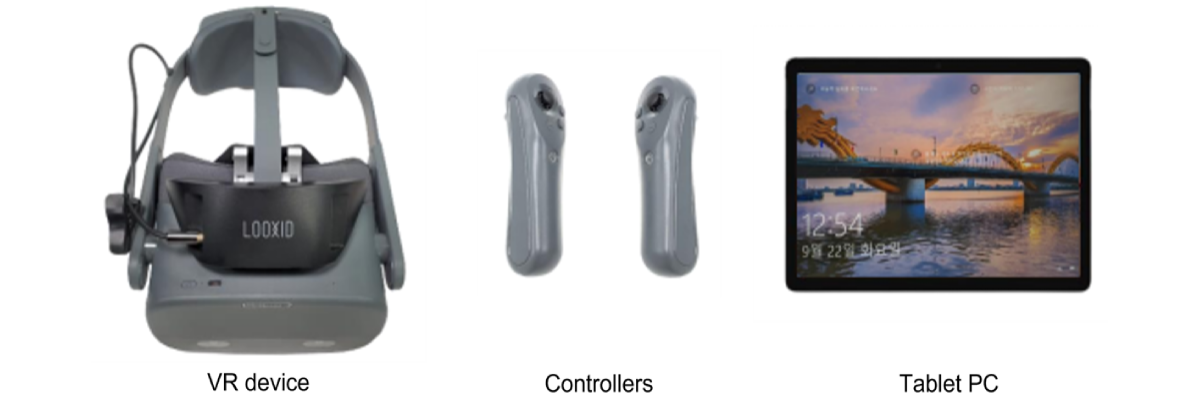
Virtual reality (VR) device system. PC: personal computer.

**Figure 3:**
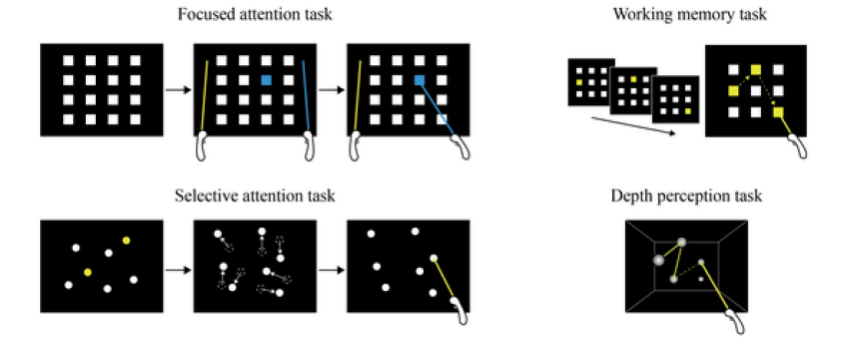
Cognitive training for each domain using virtual reality.

The VR system was used to assess the participants’ cognitive function and enhance the areas of cognitive deficiency through a VR-based cognitive training program. The assessment encompassed persistent attention, selective attention, working memory, and depth perception, all of which were conducted in a VR environment (Figures 2 and 3).

#### Immediate Object Location

The visual cognitive assessment test was conducted in a VR environment and comprised 2 phases: memory and recall. During the memory phase, participants were instructed to freely explore a 3D space and memorize the types and locations of objects within that space. Subsequently, in the recall phase, the participants were presented with a scene in which some of the objects from the memory phase had disappeared, and their challenge was to identify and reposition the objects they had observed in their original locations.

##### Recall

After a cognitive evaluation involving the adaptation of the Corsi block-tapping test to VR, participants were asked to recall a sequence of cubes displayed on the screen as they lit sequentially [13]. This task included both forward recall, in which participants remembered the exact order of cube activation, and backward recall, in which they recalled the reverse order of cube activation.

##### Text Reading

As part of the cognitive evaluation, 2 passages were presented to analyze participants’ reading patterns. Participants were free to read and interact with the passages.

##### Tricky Ball

In the cognitive evaluation involving multiple balls of the same shape, several balls were illuminated with different colors. Subsequently, the balls were blinked and moved about, and the participants were tasked with tracking the balls that were illuminated in different colors without missing any. Participants then transitioned to sessions in both 2D and 3D settings to observe any differences between the 2 and select the illuminated balls while at rest.

##### Depth Perception

Participants were required to make selections based on the order of proximity to the balls dispersed in space or the order of proximity to the balls as perceived in terms of depth. The outcomes of these selections were examined.

##### Delayed Object Location

**Figure 4:**
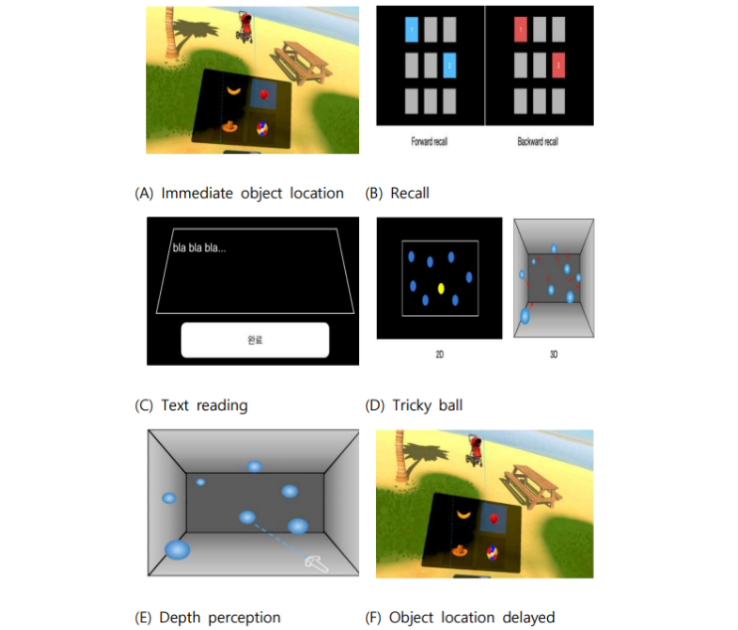
Cognitive training using virtual reality: (A) immediate object location, (B) recall, (C) text reading, (D) tricky ball, (E) depth perception, and (F) object location delayed.

Building on the cognitive evaluation conducted immediately after the object location assessment, the participants were tasked with identifying items that were not present in the initial beach scene (Figure 4).

#### VR-Based Individual Cognitive Training Program

The VR-based cognitive training program was administered for 30 minutes per individual over 24 sessions. It was specifically designed to prevent dementia and enhance cognitive function [14,15]. VR-based cognitive training programs encompass 3 categories: attention and executive function, memory, and visuospatial function.

- Busan Seagull Content: This program focuses on improving attention and executive functions. Participants were trained to spot crows flying amid seagulls.
- Firework Content: Geared toward enhancing working memory, this program tasked individuals with recalling a sequence in which fireworks exploded.
- Changed Love-House Content: Designed to enhance spatial memory, this program involved individuals in finding items within a changing room.
- Coupled Seagull Content: To improve visuospatial skills, this program challenged participants to simultaneously catch birds with both hands.
- Bird’s Poo Seagull Content: Targeting visuospatial function improvement, which required participants to catch crows with one hand while shielding themselves against flying bird excrement.

**Figure 5:**
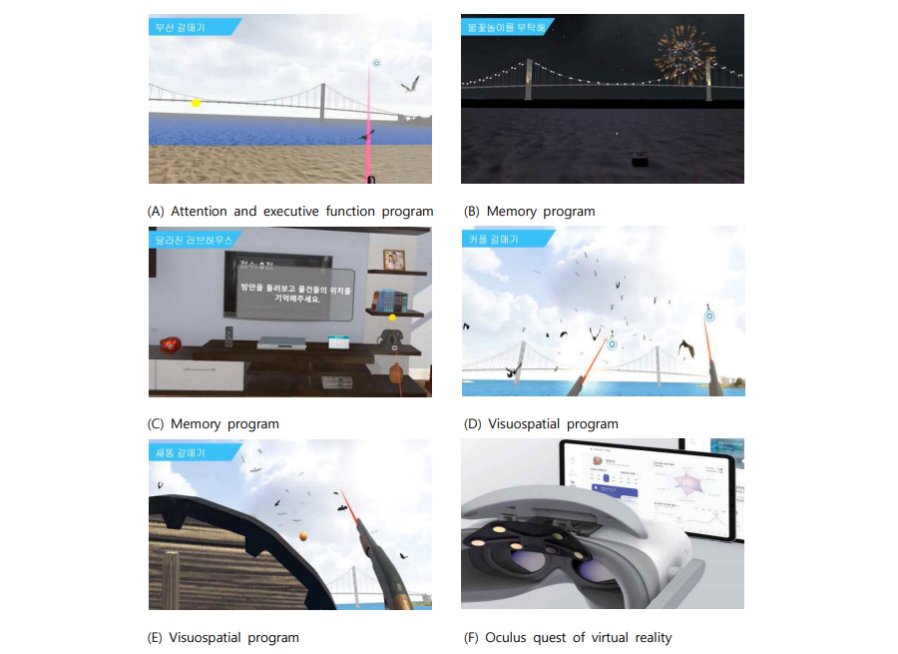
Cognitive training programs using virtual reality: (A) attention and executive function program, (B) memory program, (C) memory program, (D) visuospatial program, (E) visuospatial program, and (F) oculus quest of virtual reality.

The VR-based cognitive training program was personalized to match each participant’s cognitive function by adjusting the difficulty level accordingly (Figure 5).

#### Cognitive Function

Cognitive function was assessed using the MMSE-2nd edition, adapted for use with older adults in Korea, based on the original MMSE developed by Folstein et al [16] and Kang et al [17]. K-MoCA was created by Kang et al [18] as a quicker alternative to the MoCA developed by Nasreddine et al [19]. The DST is a component of the Korean Wechsler Adult Intelligence Scale, which assesses short-term auditory memory and attention [20]. The K-CWST measures the ability to suppress automatic responses, selectively allocate attention, and shift between tasks, thus evaluating executive function [21].

#### Depression Function

Depression was assessed using the SGDS-K, an adaptation designed to evaluate depression in older adults based on the Geriatric Depression Scale, originally developed by Yesavage et al [22] and Cho et al [23].

### Statistical Analysis

SPSS (version 22.0; IBM Corp) was used for statistical analysis of all data. Descriptive statistics, including mean (SD), were used to summarize the dependent variables within each group. A frequency analysis was conducted using descriptive statistics to compare the general characteristics of the study participants. The general characteristics of the participants were analyzed using an independent *t* test for continuous variables. A paired *t* test was used to assess changes in cognitive function and depression status in middle-aged women before and after the implementation of the VR-based individual cognitive training programs. Additionally, we conducted a repeated 2-way ANOVA to ascertain the average differences between the 2 groups. The significance level was set at *P*<.05 in all analyses.

### Ethical Considerations

This study was approved by the institutional review board of Inje University (approval number 2021-05-062-005).

## Results

### Participants’ General Characteristics

The study consisted of female participants, with a mean age of 55.17 (SD 5.81) years in the training group and 55.53 (SD 5.66) years in the control group. In the training group (n=30), 4 (13%) participants had diabetes, whereas in the control group (n=30), 2 (7%) participants had diabetes. High blood pressure was observed in 5 (17%) participants in the training group and 6 (20%) participants in the control group. Education levels averaged 12.7 (SD 2.65) years in the training group and 12.63 (SD 2.87) years in the control group. The selection criteria, as measured by the MMSE scores, were mean 26.8 (SD 1.16) points in the training group and mean 26.1 (SD 1.56) points in the control group. Notably, there were no significant differences in general characteristics between the 2 groups (Table 2). Regarding the K-MoCA scores, the training group averaged 24.87 (SD 2.62) points, while the control group averaged 24.1 (SD 1.75) points. In SGDS-K, the training group scored a mean of 3.97 (SD 2.51) points, while the control group scored a mean of 3.17 (SD 2.38) points. The homogeneity test conducted between the training and control groups revealed no significant differences (Table 3).

**Table 2:** Baseline sociodemographic characteristics.

Characteristics	Control (n=30)	Intervention (n=30)	*P* value^a^
Age (years), mean (SD)	55.53 (5.66)	55.17 (5.81)	.81
Alcohol consumed, n (%)	13 (43)	11 (37)	.61
Smoking, n (%)	0 (0)	0 (0)	—^b^
Diabetes mellitus, n (%)	2 (7)	4 (13)	.40
Hypertension, n (%)	6 (20)	5 (17)	.74
Education levels (years), mean (SD)	12.63 (2.87)	12.7 (2.65)	.93
MMSE^c^ (points), mean (SD)	26.1 (1.56)	26.8 (1.16)	.05

^a^*P*<.05 is considered significant.

^b^Not applicable.

^c^MMSE: Mini-Mental State Examination.

**Table 3:** Baseline cognitive function and depression.

Function (points)	Control (n=30), mean (SD)	Intervention (n=30), mean (SD)	*P* value^a^
K-MoCA^b^	24.1 (1.75)	24.87 (2.62)	.19
DST^c^ forward	6.53 (1.14)	6.97 (1.1)	.14
DST backward	4.4 (1.22)	4.1 (0.71)	.25
K-CWST WR^d^	111.37 (1.1)	111.77 (0.57)	.08
K-CWST CR^e^	105.37 (9.68)	105.9 (9.07)	.83
SGDS-K^f^	3.17 (2.38)	3.97 (2.51)	.21

^a^*P*<.05 is considered significant.

^b^K-MoCA: Korean version of the Montreal Cognitive Assessment.

^c^DST: Digit Span Test.

^d^K-CWST WR: Korean-Color Word Stroop Word Reading Test.

^e^K-CWST CR: Korean-Color Word Stroop Word Color.

^f^SGDS-K: Korean version of the Short Form of Geriatric Depression Scale.

### Results of Analysis of Differences in Cognitive and Depression Function Before and After Intervention

Changes in cognitive function and depression before and after the VR-based individual cognitive training programs are presented in Table 4. A comparison of the mean scores before and after K-MoCA revealed a significant increase from 24.87 (SD 2.62) to 27.50 (SD 1.70) in the training group and from 24.10 (SD 1.75) to 24.50 (SD 2.67) in the control group. Similarly, when comparing the average scores before and after DST forward, a significant difference was observed, as the mean scores increased from 6.97 (SD 1.10) to 7.90 (SD 1.18) in the training group and from 6.53 (SD 1.14) to 6.60 (SD 1.16) in the control group. The same pattern was observed for DST backward, where the mean scores increased significantly from 4.10 (SD 0.71) to 4.77 (SD 1.22) in the training group and from 4.40 (SD 1.22) to 4.23 (SD 0.86) in the control group. In contrast, while the mean scores before and after K-CWST WR increased slightly from 111.77 (SD 0.57) to 111.97 (SD 0.18) in the training group and from 111.37 (SD 1.10) to 111.73 (SD 0.64) in the control group, no significant difference was detected. Likewise, no significant difference was observed in the average scores before and after K-CWST CR, with the mean scores increasing from 105.90 (SD 9.07) to 107.80 (SD 8.61) in the training group and from 105.37 (SD 9.68) to 107.20 (SD 7.96) in the control group. However, a significant difference was identified in the mean scores before and after SGDS-K, as they decreased from 3.97 (SD 2.51) to 2.13 (SD 1.87) in the training group and from 3.17 (SD 2.38) to 3.03 (SD 1.35) in the control group, signifying a notable reduction in depression.

**Table 4:** Changes in measurements of cognitive function and depression at baseline and after 12 weeks in the training and control groups.

Variables and groups			Pretest, mean (SD)	Posttest, mean (SD)		Difference		*t* test (*df*)			*P* value^a^	
**K-MoCA^b^ (points)**												.01
	Control	24.1 (1.75)		24.5 (2.67)	0.4		–1.379 (29)			.18		
	Intervention	24.87 (2.62)		27.5 (1.7)	2.63		–5.046 (29)			.001		
**DST^c^ forward (points)**												.01
	Control	6.53 (1.14)		6.6 (1.16)	0.07		–0.403 (29)			.69		
	Intervention	6.97 (1.1)		7.9 (1.18)	0.93		–4.157 (29)			.001		
**DST backward (points)**												.01
	Control	4.4 (1.22)		4.23 (0.86)	–0.17		0.841 (29)			.41		
	Intervention	4.1 (0.71)		4.77 (1.22)	0.67		–3.247 (29)			.003		
**K-CWST WR^d^ (points)**												.41
	Control	111.37 (1.1)		111.73 (0.64)	0.36		–2.164 (29)			.04		
	Intervention	111.77 (0.57)		111.97 (0.18)	0.2		–1.795 (29)			.08		
**K-CWST CR^e^ (points)**												.96
	Control	105.37 (9.68)		107.2 (7.96)	1.83		–1.515 (29)			.14		
	Intervention	105.9 (9.07)		107.8 (8.61)	1.9		–2.419 (29)			.02		
**SGDS-K^f^ (points)**												<.001
	Control	3.17 (2.38)		3.03 (1.35)	–0.14		0.403 (29)			.69		
	Intervention	3.97 (2.51)		2.13 (1.87)	–1.84		5.061 (29)			.001		

^a^*P*<.05 is considered significant.

^b^K-MoCA: Korean version of the Montreal Cognitive Assessment.

^c^DST: Digit Span Test.

^d^K-CWST WR: Korean-Color Word Stroop Word Reading Test.

^e^K-CWST CR: Korean-Color Word Stroop Word Color.

^f^SGDS-K: Korean version of the Short Form of Geriatric Depression Scale.

## Discussion

This study investigated the impact of VR-based individual cognitive training programs on cognitive function and depressive symptoms in middle-aged women at risk of cognitive impairment. After the VR-based cognitive training programs conducted before and after this study, the scores in the training group showed a significant improvement compared with those in the control group on both the K-MoCA and DST assessments, confirming a noteworthy difference.

Previous research suggests that VR-based cognitive training is effective in delaying or preventing dementia among middle-aged individuals at high risk of developing the condition. This efficacy can be attributed to its ability to strengthen the cognitive reserve in the brain and stimulate neuroplasticity [24,25]. Moreover, VR-based cognitive training interventions are particularly suited to enhance executive functions such as attention and memory [26]. The VR-based cognitive training program used in this study included attention and executive function, work and spatial memory, and visuospatial ability training. Consequently, consistent participation in cognitive training contributes to the overall improvement in cognitive function.

Conventional rehabilitation training can become monotonous and uninspiring, potentially reducing the participants’ ability to concentrate on training. Conversely, game-based cognitive training, as implemented in this VR program, effectively bolsters concentration by making the training more engaging and enjoyable.

In addition, the VR measurement system used for VR-based cognitive evaluations provides immediate visual feedback on cognitive function scores and areas of cognitive deficiency, thereby enhancing immersion and motivation within the VR environment. This domain-specific cognitive training intervention is believed to play a pivotal role in improving cognitive function.

Visual and auditory attention are essential for performing cognitive tasks. VR-based cognitive training interventions enhance connectivity between the frontal and occipital brain regions by engaging in temporal and spatial functions, resulting in heightened realism, presence, and immersion [27]. Consequently, cognitive training that consistently stimulates these interconnected brain regions through a visual, auditory, or spatial multisensory approach within a VR environment is considered a crucial strategy to prevent cognitive function decline.

In the SGDS-K test, conducted to evaluate depressive symptoms after the intervention, scores in the training group exhibited a significant reduction compared with those in the control group, indicating a substantial difference. Previous research has demonstrated that behavioral activation and physical activity can lead to a reduction in depressive symptoms [28].

In this study, HMDs were used to induce immersion by aligning head movements in the direction of gaze. Furthermore, the continuous upper-limb movement facilitated by the controller expands the range of motion, contributing to increased physical activity. VR technology offers immediate feedback, fosters enjoyment, and piques interest, thereby enhancing the participants’ motivation for rehabilitation. Consequently, the competitive and cooperative dynamics observed among participants during gaming activities were effective in reducing depression as they promoted motivation.

The VR-based cognitive training program, designed as an engaging gaming experience in a virtual environment, is expected to motivate participants by immersing them in an alternative reality. This approach encourages physical activity and behavior, potentially alleviating the symptoms of depression.

### Limitations

This study has some limitations. First, it focused exclusively on women, and the sample size was relatively small, limiting the generalizability of the results. Moreover, individual variations in cognitive aging among middle-aged women do not account for all potential factors contributing to dementia risk. Conditions such as high blood pressure and diabetes can negatively affect various cognitive functions, including reaction time, processing speed, and working memory evaluation [29], and are known risk factors for dementia in middle-aged women. Additionally, factors such as increased weight, abdominal obesity, reduced physical strength, and poor overall health are associated with dementia risk [30,31]. Future research should aim to establish clinical significance by including a larger and more diverse participant pool while also developing interventions that take into account multiple risk factors for dementia. Furthermore, exploring whether VR-based cognitive training programs can effectively manage these risk factors in middle-aged women at risk of cognitive impairment could lead to more effective early prevention strategies.

### Conclusions

This study aimed to examine the impact of VR-based individual cognitive training programs on cognitive function and depression in middle-aged women to address the risk of cognitive impairment. The findings of this study confirmed the effectiveness of VR-based cognitive training programs in enhancing cognitive function and reducing depression in middle-aged women at risk of cognitive impairment. This nonpharmacological cognitive training program is considered beneficial for improving cognitive function and alleviating depression in this population. This offers a promising avenue for early prevention of cognitive impairment in middle-aged women facing such risks.

## Appendix

Multimedia Appendix 1 CONSORT-eHEALTH checklist (V 1.6.1).

## Abbreviations

DST: Digit Span Test
EOAD: early-onset Alzheimer disease
HMD: head-mounted display
K-CWST: Korean-Color Word Stroop Test
K-MoCA: The Korean version of the Montreal Cognitive Assessment
MMSE: Mini-Mental State Examination
SGDS-K: The Korean version of the Short Form of Geriatric Depression Scale
VR: virtual reality
